# A DNA Vaccine Encoding the VAA Gene of *Vibrio anguillarum* Induces a Protective Immune Response in Flounder

**DOI:** 10.3389/fimmu.2019.00499

**Published:** 2019-03-19

**Authors:** Jing Xing, Hongsen Xu, Xiaoqian Tang, Xiuzhen Sheng, Wenbin Zhan

**Affiliations:** ^1^Laboratory of Pathology and Immunology of Aquatic Animals, KLMME, Ocean University of China, Qingdao, China; ^2^Laboratory for Marine Fisheries Science and Food Production Processes, Qingdao National Laboratory for Marine Science and Technology, Qingdao, China

**Keywords:** VAA DNA vaccine, *Vibrio anguillarum*, *Paralichthys olivaceus*, humoral immune response, cellular immune response, immune protection

## Abstract

*Vibrio anguillarum* is a pathogenic bacterium that infects flounder resulting in significant losses in the aquaculture industry. The VAA protein previously identified in flounder is associated with a role in immune protection within these fish. In the present study, a recombinant DNA plasmid encoding the *VAA* gene of *V. anguillarum* was constructed and its potential as a DNA vaccine, to prevent the infection of *V. anguillarum* in flounder fish, investigated. We verified the expression of the VAA protein both *in vitro* in cell lines and *in vivo* in flounder fish. The protective effects of pcDNA3.1-VAA (pVAA) were analyzed by determination of the percentage of sIgM^+^, CD4-1^+^, CD4-2^+^, CD8β^+^ lymphocytes, and the production of VAA-specific antibodies in flounder following their immunization with the DNA vaccine. Histopathological changes in immune related tissues, bacterial load, and relative percentage survival rates of flounder post-challenge with *V. anguillarum*, were all investigated to assess the efficacy of the pVAA DNA vaccine candidate. Fish intramuscularly immunized with pVAA showed a significant increase in CD4-1^+^, CD4-2^+^, and CD8β^+^ T lymphocytes at days 9, 11, and 14 post-vaccination, reaching peak T-cell levels at days 11 or 14 post-immunization. The percentage of sIgM^+^ lymphocytes reached peak levels at weeks 4–5 post-immunization. Specific anti-*V. anguillarum* or anti-rVAA antibodies were induced in inoculated fish at days 28–35 post-immunization. The liver of vaccinated flounder exhibited only slight histopathological changes compared with a significant pathology observed in control immunized fish. Additionally, a lower bacterial burden in the liver, spleen, and kidney were observed in pVAA protected fish in response to bacterial challenge, compared with pcDNA3.1 vector control injected fish. Moreover, the pVAA vaccine confers a relative percentage survival of 50.00% following *V. anguillarum* infection. In summary, this is the first study indicating an initial induction of the T lymphocyte response, followed by B lymphocyte induction of specific antibodies as a result of DNA immunization of flounder. This signifies the important potential of pVAA as a DNA vaccine candidate for the control of *V. anguillarum* infection.

## Introduction

Flounder (*Paralichthys olivaceus*) is a common fish species within the aquaculture industry in the North of China. Plans to increase fish production, for the economic benefit of local fishermen, could be severely hampered by the potential infection of the fish population with *Vibrio anguillarum*, a Gram-negative bacterium that causes considerable losses in the fishing and aquaculture industries (1). The use of vaccines for disease prevention in the flounder population is considered the most acceptable method for aquaculture, where the number of fish at risk of infection is high and therapeutic options pose practical, technical, and economic challenges (2). Moreover, vaccines confer safety advantages for both fish and the environment (3), while the use of chemicals and antibiotics are associated with the potential disadvantages of accumulation in the flesh of animals, the appearance of drug-resistant strains, and contamination of the aquatic environment (4, 5). Such vaccines targeted to *V. anguillarum*, however, are not yet available for use in China and any prospects of their development are limited by a dearth of information about the adaptive immune response of fish following vaccination.

In a previous study, we successfully identified the immunogenic VAA protein from *V. anguillarum*. Immunization of fish with recombinant VAA (rVAA) protein has been shown to give rise to a humoral immune response that confers effective immune protection against *V. anguillarum* challenge (6). The successful design of a DNA vaccine and determination of its efficacy requires that any candidate results in a potent stimulation of the immune system, either via humoral, or cellular immune responses (7, 8). The aim of this study was to construct a recombinant DNA plasmid containing the VAA gene of *V. anguillarum* and investigate any immune protection effects observed after vaccination.

In flounder, T-cell surface molecular marker genes, CD4-1, CD4-2, CD8α, and CD8β, have been cloned and their corresponding specific antibodies produced (9, 10). Numerous researchers have studied the cellular immune response resulting from DNA vaccination via analysis of the transcription levels of T-cell marker genes in spleen and head kidney (11–13). However, limited research exists concerning the potential variation in the different subsets of T lymphocyte. Work from our laboratory has previously demonstrated variations in T lymphocyte subsets following Hirame novirhabdovirus (HIRRV) infection and immunization (14). This insight prompted us to investigate the cellular immune response following DNA vaccination. Effective vaccines result in the production of antibodies and induction of sIgM^+^ B lymphocytes (15). Indeed, a previous study using *in vitro-*produced monoclonal antibodies against flounder IgM (FIgM-Mab) demonstrated the induction of sIgM^+^ lymphocytes following immunization with a DNA vaccine (13, 16).

In this study, we report the construction of a recombinant DNA plasmid encoding the *VAA* gene of *V. anguillarum*. Using this plasmid, we investigated both VAA expression *in vitro* and *in vivo*, and analyzed the immune protective effects in a flounder model. Additionally, the humoral and cellular immune responses, in terms of the production of specific antibodies and percentages of CD4-1^+^, CD4-2^+^, CD8β^+^, sIgM^+^ lymphocytes, were investigated in flounder fish to examine the potential of pVAA as a DNA vaccine in the maintenance of aquaculture fish populations.

## Materials and Methods

### Construction of DNA Vaccine Plasmid

The pcDNA3.1 plasmid containing a cytomegalovirus (CMV) promoter, the bovine growth hormone (BGH) polyadenylation signal for transcription termination, the Ampicillin resistance gene, and the ColE1 origin of replication for maintenance in *Escherichia coli*, was used as the vector to construct the recombinant plasmid pcDNA3.1-VAA (pVAA). Briefly, the *VAA* gene (GeneBank accession number WP_013857004.1) was obtained by PCR with *VAA* specific primers (Table 1). The amplified *VAA* gene was digested with *Kpn* I and *Eco*R I and then integrated into the pcDNA3.1 plasmid, resulting in the pVAA plasmid (Supplementary Figure 1A). The sequence of the recombinant plasmid was verified by specific PCR amplification and DNA sequencing, and then digested with *Kpn* I and *Eco*R I to confirm the appropriate insertion of *VAA* gene into pcDNA3.1 (Supplementary Figure 1B). The DNA vaccine plasmid was extracted using an EndoFree plasmid Kit (Tiangen, Beijing, China) according to the manufacturer's instructions, and the concentration measured using a Nanodrop 8000 Spectrophotometer (ThermoFisher, Waltham, MA, USA). Subsequently, it was dissolved in sterile water to a final concentration of 500 ng/μl for transfection and suspended in sterile PBS at a final concentration of 200 ng/μl for immunization, before being stored at −20°C until use.

**Table 1:** Primers used in this paper.

**Primer name**	**Primer sequence (5′-3′)**	**Source**
VAA-F	GGGGTACCACCATGAACAGTACTTTTATCGTC (*Kpn* I)	WP_013857004.1
VAA-R	CGGAATTCTTACACTTCTAATATCACGCG (*EcoR* I)	
18S-F	GGTCTGTGATGCCCTTAGATGTC	EF126037
18S-R	AGTGGGGTTCAGCGGGTTAC	
rpoS-F	GAAGATGCCAAAGAAGGGTTT	VAA_RS12590
rpoS-R	GAGCATTTGCGTACTAGCTTT	

*The underlined letters represent the restriction enzyme sites*.

### Antibodies, Cells, Bacteria, And Animals

The rVAA protein was expressed and purified from *E. coli*, before being used for polyclonal antibody production in mice, as previously reported (6, 17). Western blot analysis revealed that the resulting antiserum specifically bound the purified rVAA, confirming the specificity of produced polyclonal antibodies (Supplementary Figure 2). The mouse polyclonal antibodies were diluted 1:1000 with PBS and used in immunofluorescence assay (IFA) and flow cytometry (FCM). Monoclonal antibodies against flounder IgM (FIgM-Mab) were produced by our laboratory, diluted 1:1,000 with PBS, and used in FCM, western blot, and enzyme-linked immunosorbent assays (ELISA) as previously reported (14). The rabbit anti-flounder CD4-1, CD4-2, and CD8β polyclonal antibodies (FCD4-1-Pab, FCD4-2-Pab, FCD8β-Pab) were also produced in our laboratory, and diluted to 1:1,000, 1:1,500, and 1:2,000 with PBS, for use in FCM assays, as previously reported (14).

HINAE cells, generously provided by Dr. Ikuo Hirono of Tokyo University of Marine Science and Technology (13), were seeded in 6-well plates, cultured in Leibovitz's L-15 medium containing 20% FBS, 100 IU/ml penicillin, and 100 μg/ml streptomycin, and used for transfection to analyze the transcription and translation of the *VAA* gene by RT-PCR, FCM, and IFA.

*V. anguillarum* was isolated and stored in our laboratory (18). The bacteria were cultured at 37°C with Luria Bertani (LB) medium for 12 h, harvested by centrifugation at 8,000 × g for 5 min, and the concentration measured using an Accuri C6 cytometer (BD Biosciences, Piscataway, NJ, USA). Bacteria were subsequently used for challenge and in ELISA at a concentration of 1.0 × 10^7^ CFU/ml.

Healthy flounder (*P. olivaceus*, 35 ± 5 g), purchased from a fish farm in Rizhao, Shandong province, China, were confirmed free of *V. anguillarum*, as described previously (18), before being used for immunization and challenge experiments. Fish were raised at 21 ± 0.5°C, fed with food pellets twice a day, and anesthetized with MS-222 prior to immunization, challenge, and sampling.

### Vaccination, Sampling, and Challenge

Three hundred flounder were randomly divided into pVAA and pcDNA3.1 control groups (150 fish/group). After acclimation for 1 week, fish were intramuscularly injected with 20 μg pVAA or pcDNA3.1 in the epaxial muscle below the dorsal fin. At day 7 post immunization, fish were sacrificed and bled through the caudal vein prior to the sampling of muscle tissues. Tissues (0.5–1.0 cm^3^) surrounding the site of injection were extracted, embedded in tissue freezing medium (Leica Biosystems, Wetzlar, UK) and immediately frozen at −80°C, prior to the analysis of VAA expression by IFA.

For the detection of sIgM^+^ B lymphocytes, the lymphocytes in peripheral blood, spleen, and head kidney were randomly sampled from three immunized fish at weeks 3, 4, 5, 6, and 7 post-immunization. For detection of CD4-1^+^, CD4-2^+^, and CD8β^+^ T lymphocytes, nine fish were randomly sampled in each group at days 3, 5, 7, 9, 11, 14, and 28 post-immunization. The lymphocytes were isolated from peripheral blood, spleen, and head kidney by Percoll gradient density as previously described (16), before FCM analysis.

For serum isolation, blood was sampled from the caudal veins (*N* = 3) at days 28 and 35 post-inoculation, and clotted at 4°C overnight. The serum was obtained by centrifugation at 3,000 × g for 10 min and stored at −20°C before use.

For *V. anguillarum* challenge studies, 30 immunized fish were randomly selected from each group, cultured in three tanks, and bacteria administered intraperitoneally with a lethal dose of 1.0 × 10^6^ CFU (10 × LD50) live *V. anguillarum* per fish at week 6 post-immunization. Survival of each group was monitored over a period of 15 days after the challenge, and relative percent survival rate (RPS) was calculated according to the method of Amend et al. (19). Meanwhile, liver, kidney, and spleen were sampled at day 7 post-challenge and used for histopathology and qPCR analyses.

### Immunofluorescence Analysis

IFA was performed to visualize the expression of VAA protein in transfected HINAE cells and inoculated fish.

HINAE cells were seeded on coverslips and transfected with 500 ng of DNA plasmid using Lipofectamine^®^ 3000 (Thermo-Fisher, Boston, MA, USA). Two days after transfection, cells were fixed with 4% (w/v) paraformaldehyde for 15 min, washed three times with PBS and permeabilized with 0.1% (v/v) Triton X-100 for 10 min. Non-specific binding was blocked with 3% (v/v) BSA at 37°C for 1 h. The slides were then washed three times with PBS and subsequently incubated with mouse anti-rVAA antibody at 37°C for 1 h. The cells were washed three times as described above and incubated with Alexa Fluor 488-conjugated goat anti-mouse immunoglobulin G (IgG) (Thermo-Fisher) at 1:1,000 dilutions at 37°C for 1 h. After washing three times with PBS, 4′, 6-diamidino-2-phenylindole (DAPI, 1:1,000, Thermo-Fisher) was used to stain the nuclei of all cells at room temperature for 10 min. The coverslip-bound cells were washed again before being mounted onto slides and observed under an epifluorescence microscope IX71 (Olympus, Tokyo, Japan). Cells transfected with pcDNA3.1 were used as a negative control.

Muscle tissue from around the injection site (0.5–1.0 cm^3^) was taken seven days post-immunization and cryo-sectioned (7 μm in thickness) with a Leica CM 1900 microtome (Leica). The expression of VAA was analyzed by IFA. Briefly, sections were transferred to microscope slides treated with poly-L-lysine, fixed with precooled acetone for 10 min, air-dried for 15 min, and then blocked with 3% BSA overnight at 4°C. The slides were subsequently incubated with mouse anti-rVAA polyclonal antibody and goat anti-mouse IgG-488 at 37°C for 1 h each, and then stained by DAPI (1:1,000) for 10 min in a moisture chamber. Finally, the microscope slides were washed 3 times with PBS and mounted in antifade solution for observation using a fluorescent microscope IX71 (Olympus). Muscles from pcDNA3.1 injected fish were used as a negative control.

### RT-PCR and qPCR

For *in vitro* VAA transcription studies, HINAE cells, at ~70–80% confluence, were transfected with 500 ng of pVAA or pcDNA3.1. Transfected cells were cultured at 24°C for 48 h prior to being placed in RNA later reagent (TaKaRa, Tokyo, Japan). Total RNA was extracted using TRIZOL reagent (Baosheng, Dalian, China) and the concentration of RNA measured using a Nanodrop 8000 Spectrophotometer (Thermo-Fisher). Complementary DNA (cDNA) was synthesized using a Reverse Transcriptase M-MLV kit (TaKaRa) according to the manufacturer's instructions and was subsequently used as the template for PCR amplification. The 18S rRNA gene was used as an internal control. The specific primers used in this study are listed in Table 1.

To quantify the bacterial load post-challenge, *rpoS* (GenBank: AY695433), a housekeeping gene for the general regulation of stress, was selected as the target gene and qPCR carried out as described previously (20, 21). Briefly, bacterial DNA (10 μg) was extracted from 2.0 × 10^8^ CFU of *V. anguillarum* using a TIANamp Bacteria DNA Kit (Tiangen, Beijing, China). Bacterial DNA was serially diluted over a 10-fold dilution range, corresponding to 2.0 × 10^8^-2.0 × 10^4^ bacterial cells, in order to generate the standard curve between Ct values and bacterial load. The logarithm of *V. anguillarum* load was calculated following the linear equation:

$$\begin{array}{l} \text{Y}=\text{A}\times \text{X}\pm \text{B }\left(\text{A}:\text{ slope};\text{B}:\text{ intercept}\right) \end{array}$$

In order to quantify the bacterial burden in immunized flounder, tissues (liver, spleen, and kidney) from pVAA- and pcDNA3.1-immunized fish were sampled at day 7 post-challenge. The whole genome was extracted from the tissues of analysis (0.1 g) using the TIANamp DNA Kit (Tiangen, Beijing, China) according to the manufacturer's protocol. DNA samples were used as a template for further qPCR amplification, which was performed using a LightCycler^®^ 480 real-time PCR system (Roche Life Science, USA). Ct values were obtained and converted into *V. anguillarum* counts according to the formula of the standard curve. Samples from healthy flounder were used as negative controls, with all experiments performed in triplicate.

### Flow Cytometry

The ratio of cells expressing VAA was investigated by FCM. Briefly, 10^6^ pVAA-transfected cells or pcDNA3.1 transfected controls were harvested by digestion with pancreatin at 48 h post-transient transfection. Cells were probed with mouse anti-rVAA antibodies (1:1,000) and stained with goat anti-mouse IgG-488 secondary antibodies (1:1,000). Afterward, the cell suspensions were analyzed using the Accuri C6 cytometer (BD Biosciences, Piscataway, NJ, USA). Ten thousand cells per sample were analyzed using a forward scatter filter (530 nm) and percentage green fluorescent cells analyzed using the C6 Flow Plus (BD, USA) program.

To investigate the variation of sIgM^+^, CD4-1^+^, CD4-2^+^, or CD8β^+^ lymphocytes in peripheral blood, spleen, and head kidney from flounder fish, FCM was performed as previously reported (14). Briefly, to determine the percentage of sIgM^+^ lymphocytes in the fish tissues, cells isolated from peripheral blood, spleen, and head kidney were counted and adjusted to 1 × 10^6^ cells/ml, then incubated with mouse anti-IgM monoclonal antibody for 1 h at 37°C. Subsequently, cells were washed three times with PBS, incubated with goat-anti-mouse Ig-FITC (1:256 diluted in PBS, Sigma, St. Louis, MO, USA) at 37°C for 1 h, and washed with PBS again. For the detection of CD4-1^+^, CD4-2^+^, or CD8β^+^ lymphocytes, the lymphocytes extracted from each fish were incubated with FCD4-1-Pab, FCD4-2-Pab, or FCD8β-Pab, respectively, and then incubated with Fluor 647-conjugated goat anti-rabbit IgG (1:1,000, Thermo Fisher Scientific, USA). Cell suspensions were further analyzed using the Accuri C6 cytometer (BD Biosciences, Piscataway, NJ, USA) as previously reported (14). Briefly, lymphocytes with similar cell granularity and cell size, as determined by side- and forward-scatter (SSC and FSC) parameters, respectively, were gated in the R1 scope of the dot plots. The percentage of cells distributed among R1 was analyzed using fluorescent histograms in which FITC-labeled cells and Alexa Fluor® 647 labeled cells were captured by fluorescent light (FL)-1 and FL-4, respectively. Myeloma culture supernatant or rabbit negative serum were used as negative controls, in place of the aforementioned primary antibodies.

### ELISA

The ability of the pVAA vaccine to induce a specific antibody response in flounder was evaluated by indirect ELISA. Briefly, 10^6^ CFU *V. anguillarum* or 10 μg rVAA per well were coated in 96-well microplates (Corning Life Science, New York, NY, USA) at 4°C overnight. After blocking with 5% BSA in PBS at 37°C for 1 h, the plates were incubated with a series of diluted flounder anti-serum (1:32, 1:64, 1:128, 1:256, 1:512, 1:1,024, and 1:2,048) at 37°C for 1 h. The plates were washed three times with PBS-T and incubated with flgM. Following further washing, alkaline phosphatase (AP)-conjugated goat-anti-mouse IgG (1:5,000, Sigma) was added and incubated for 1 h at 37°C. Finally, 100 μl 0.1% (w/v) p-nitrophenyl phosphate (pNPP, Sigma, St. Louis, MO, USA) in 50 mM carbonate-bicarbonate buffer (pH 9.8) containing 0.5 mM MgCl_2_ was added to each well. The absorbance was measured using an automatic ELISA reader at 405 nm. All serum samples were assayed in triplicate. Endpoint titers were the corresponding ultimate dilutions at which the P/N > 2.1 (P: absorbance from pVAA immunized fish serum; N: absorbance from pcDNA3.1 immunized fish serum). Serum from pcDNA3.1 injected fish was used as a negative control, to substitute for anti-VAA antibodies.

### Western Blot

Western blot was used to analyze the immunospecificity of anti-sera from vaccinated fish. Briefly, rVAA protein was electrophoresed by 12% sodium dodecyl sulfate-polyacrylamide gel electrophoresis (SDS-PAGE) and electrically transferred onto poly-vinylidene fluoride (PVDF) membranes. The membranes were blocked with 3% BSA overnight at 4°C and washed three times with PBS-T. Subsequently, the sera (diluted at 1:100) from pVAA injected fish at day 35 post-vaccination, fIgM-Mab and goat-anti-mouse IgG-AP (1:4,000, Sigma), were used as primary, secondary, and tertiary antibodies, respectively, and incubated for 1 h at 37°C. Samples were washed three times with PBS-T, and positive bands detected by incubating membranes with prepared substrate solution (100 mM NaCl, 100 mM Tris, and 5 mM MgCl_2_, pH 9.5) containing 5-bromo-4-chloro-3-indolyphosphate (BCIP, Sigma) and nitroblue tetrazolium (NBT, Sigma) for 5 min, and reactions stopped with distilled water. Sera from pcDNA3.1 injected fish were used as primary antibody negative controls.

### Histopathology

Spleen, kidney, and liver of flounder fish, at day 7 post-challenge, were removed for histopathology analysis by hematoxylin-eosin (H&E) staining. Samples were fixed in Bouin's fluid for 18 h, washed free of Bouin's fluid with 70% alcohol, and embedded in paraffin. The tissues were then sectioned into 7 μm thick sections, transferred onto pretreated microscope slides, and dried at 37°C overnight. After deparaffinization with xylene and 50% xylene ethanol solution, sections were rehydrated by successively immersing in a series of 95%, 80%, 70%, 50%, and then 30% ethanol for 4 min each. Nuclear structures were stained with hematoxylin for 10 min, differentiated in 0.1% acid alcohol for 45 s, and washed for 30 min with water. Subsequently, the sections were dehydrated through a gradient ethanol solution, counterstained for cytoplasmic structures using eosin for 45 s, and differentiated in 95% ethanol for 45 s. Finally, the slides were dehydrated with two changes of 100% ethanol, cleared in xylene, mounted using neutral balsam, and examined for histological changes with a light microscope (Olympus DP70).

### Immunohistochemistry

The presence of *V. anguillarum* in flounder liver, spleen, and kidney samples at day 7 post-challenge was detected by IHC. Briefly, the tissue sections were deparaffinized in xylene, rehydrated in a series of ethanol, and retrieved in sodium citrate buffer (10 mM, pH 6.0) by heating for 20 min and then cooled to room temperature. Endogenous peroxidase activity was inhibited by incubation with 3% hydrogen peroxide for 15 min. The slides were blocked with 3% BSA at 37°C for 1 h, then successively incubated with sera from pVAA injected fish at day 35 post-vaccination (diluted at 1:32) and fIgM-Mab for 1 h at 37°C. Slides were then rinsed in PBS before being incubated for 10 min with biotinylated goat anti-mouse IgG (1:1,000) and a dual link system-HRP. *V. anguillarum* was visualized by incubation with the chromogenic substrate diaminobenzidine (DAB) (ZSGB-BIO), following the manufacturer's instructions. Finally, slides were counterstained with hematoxylin for 15 min, washed in acid ammonia solution for 15 s, and mounted using 60% glycerin. Slides were photographed with a light microscope (Olympus DP70). Serum from healthy flounder was used as a control in place of primary antibody.

### Statistical Analysis

Statistical analysis was performed using GraphPad Prism5 (GraphPad software, Inc. San Diego, CA, USA). All data were expressed as mean ± standard deviation (SD). Differences between pVAA and pcDNA3.1 treated groups in FCM, ELISA, and qPCR were analyzed by a two-tailed unpaired *t*-test and a Mann–Whitney *U*-test. The significance of a protective effect conferred by pVAA over pcDNA3.1 was analyzed by a Log-rank test. Statistical differences between fish inoculated with pVAA compared to the pcDNA3.1 group were performed to levels of ^*^p < 0.05, ^**^p < 0.01, ^***^p < 0.001.

## Results

### VAA Protein Is Successfully Expressed in pVAA Transfected HINAE Cells

**Figure 1:**
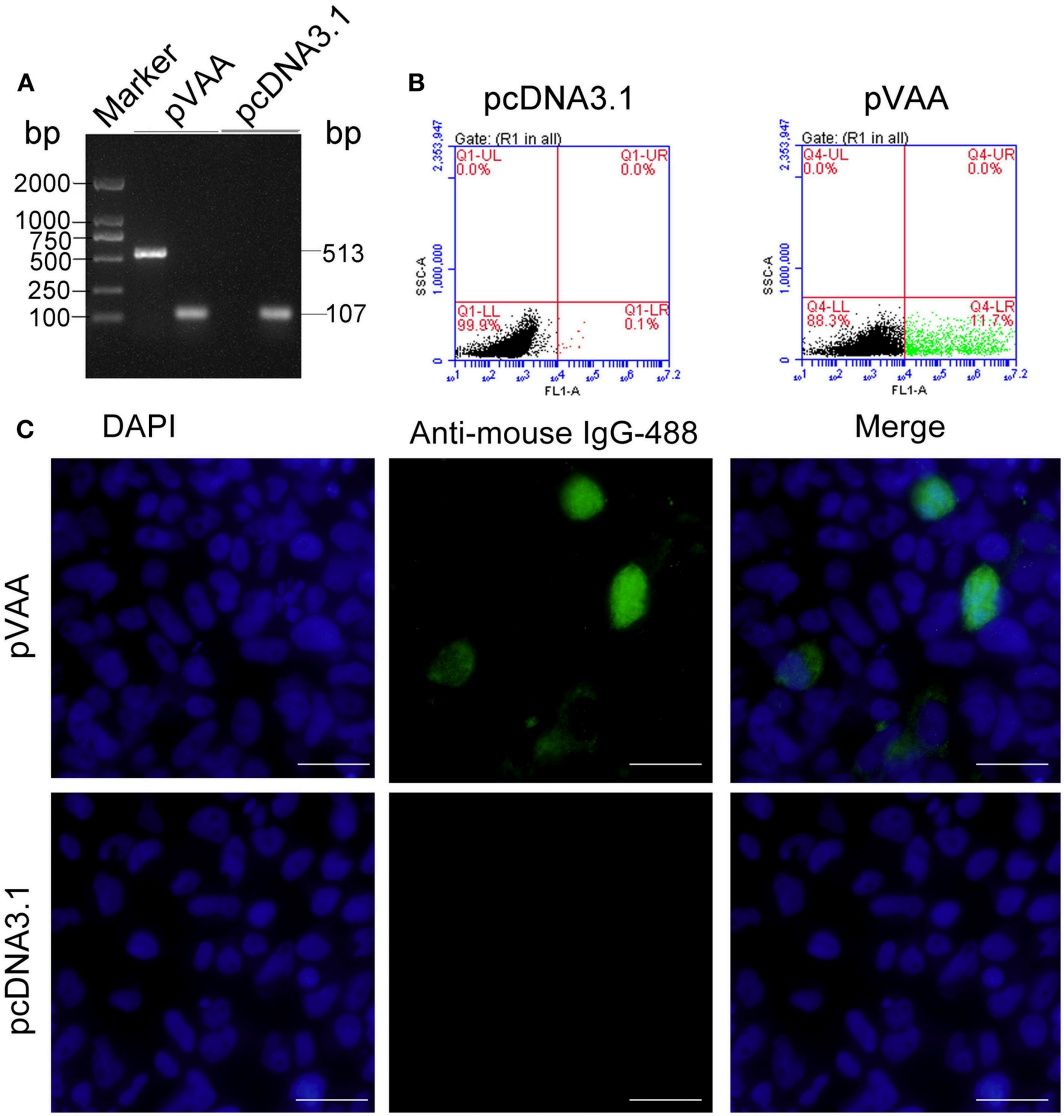
*In vitro* VAA expression in HINAE cells. **(A)** RT-PCR detection of VAA transcripts in transfected HINAE cells. **(B)** Flow cytometry analysis of cells expressing VAA protein. The cells were transfected with 500 ng of pVAA or pcDNA3.1 plasmid and stained with mouse anti-rVAA antibody. **(C)** IFA analysis for VAA protein expression in HINAE cells. 24 h after transfection, immunofluorescence labeling was performed by incubation with the mouse anti-rVAA antibody followed by the addition of goat anti-mouse IgG-488 antibody for detection. DAPI panels show control staining of cell nuclei. Scale bar, 20 μm.

RT-PCR was employed to examine the transcription of the *VAA* gene in HINAE cells. The results indicated that VAA transcripts (513 bp) could be detected in pVAA transfected cells. In contrast, no VAA transcript was observed in pcDNA3.1 control transfected cells. Control 18S rRNA transcripts were observed in both pVAA and pcDNA3.1 transfected cells (Figure 1A). The FCM results revealed that the proportion of cells that could express VAA proteins following transient transfection with pVAA was ~11.7%, while no cells expressing VAA protein were observed in the pcDNA3.1 transfected cell population (Figure 1B). Moreover, IFA analysis of HINAE cells transfected with the pVAA showed a green fluorescence specific for VAA protein expression. No fluorescence was detected in pcDNA3.1 transfected cells (Figure 1C).These results indicate that the VAA protein was successfully expressed by HINAE cells, confirming the functionality of the pVAA plasmid.

### VAA Protein Is Expressed in Flounder After Intramuscular Injection Of pVAA

**Figure 2:**
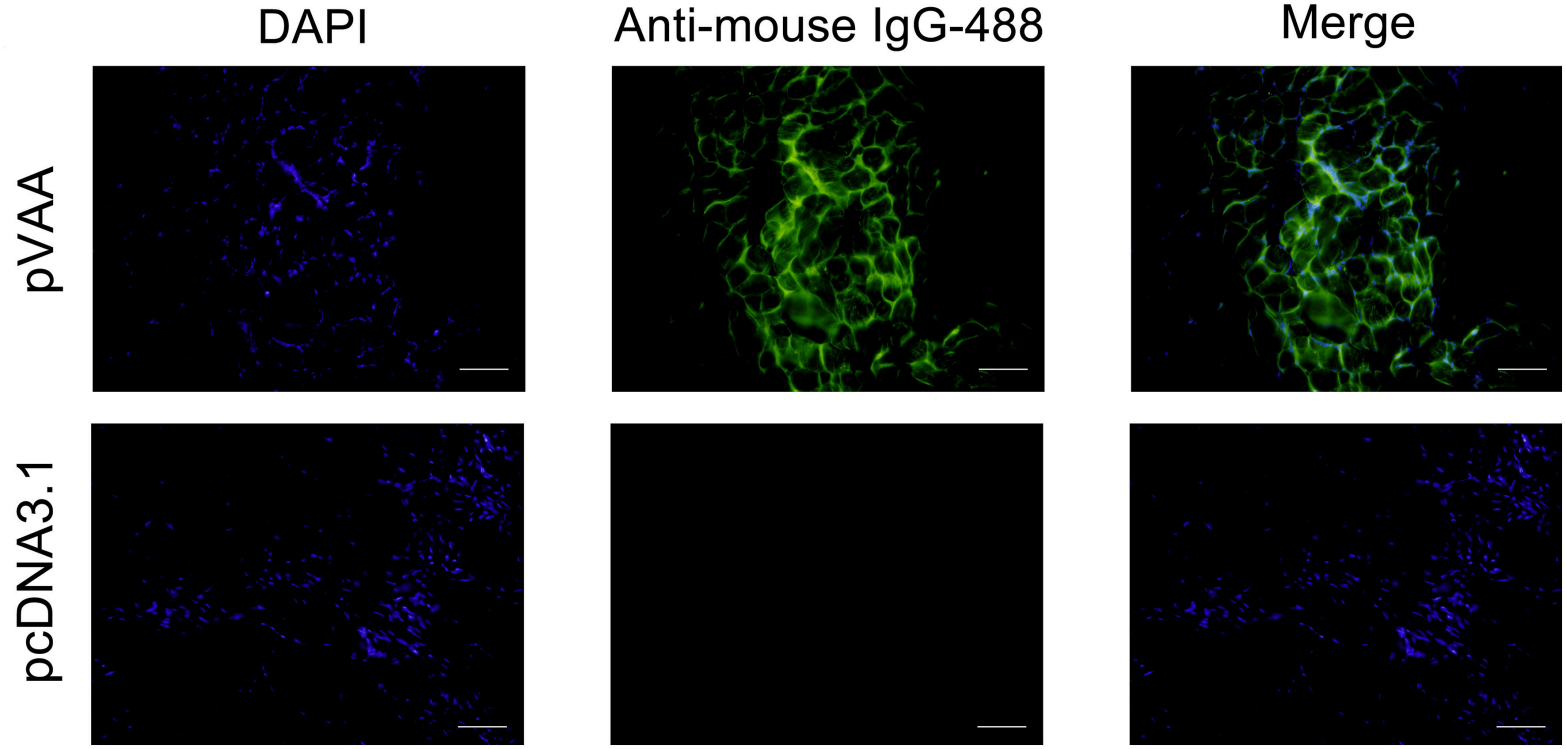
*In vivo* VAA expression in flounder fish. Muscles were excised from the injection site at day 7 post-inoculation, and cryosections (7 μm) stained with mouse anti-rVAA antibody and goat anti-mouse IgG-488 antibody to visualize the expression of VAA. DAPI panels were used to visualize cell nuclei. Scale bar: 20 μm.

To examine the expression of the VAA protein in pVAA vaccinated fish, cryosections of muscle tissue were prepared from 7 days post-immunization, and mouse anti-rVAA polyclonal antibody was used to bind to the VAA protein. A specific green fluorescence was observed in muscle sections from pVAA-inoculated fish. In contrast, no fluorescence was detected in the sections from pcDNA3.1 injected fish (Figure 2). The results revealed that the VAA protein was successfully expressed from the pVAA plasmid in vaccinated fish.

### pVAA Vaccine Induces a T-cell Immune Response

**Figure 3:**
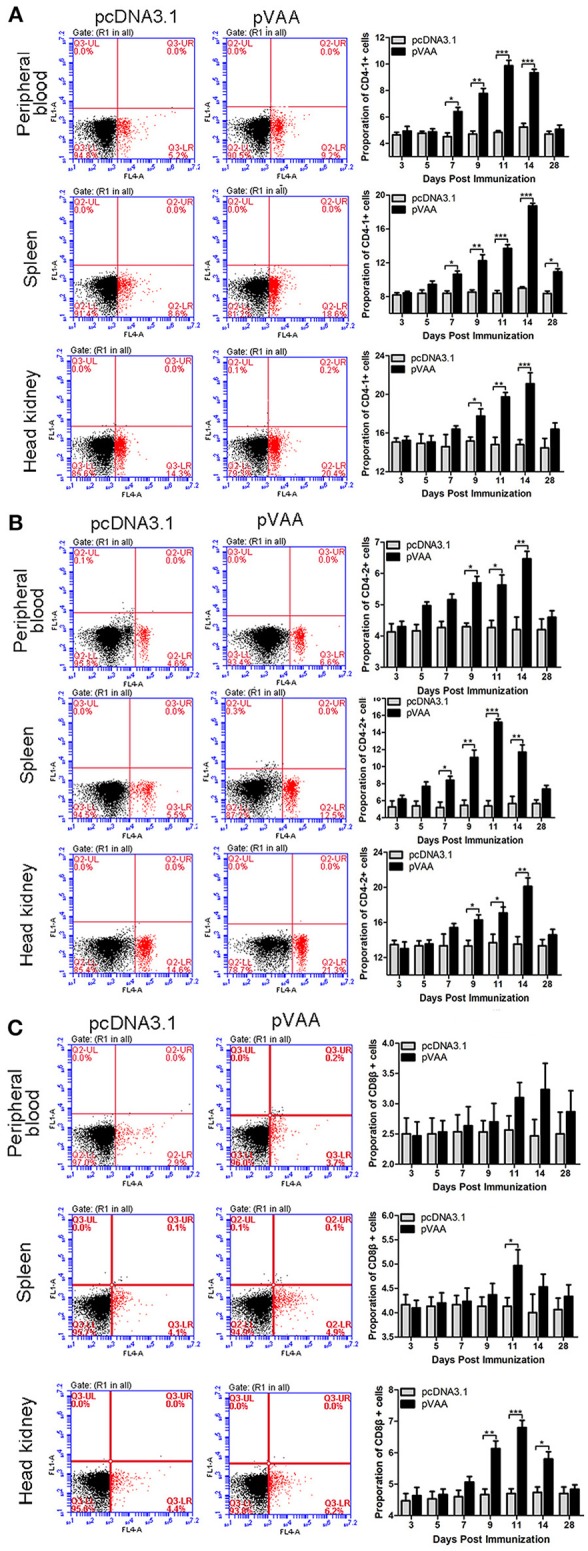
T cell immune response induced by pVAA vaccination. FCM assay investigating the percentages of **(A)** CD4-1^+^, **(B)** CD4-2^+^ and **(C)** CD8β^+^ T lymphocytes in peripheral blood, spleen, and head kidney of flounder. The plots are the results of CD4-1^+^, CD4-2^+^, and CD8β^+^ lymphocytes from pcDNA3.1 control and pVAA groups at day 14 post-immunization. Lymphocytes were isolated from immune related tissues at day 3, 5, 7, 9, 11, 14, and 28 post-immunization, and incubated with FCD4-1-Pab, FCD4-2-Pab, and FCD8β-Pab, respectively. FCM was used to analyze the stained cells. All values are expressed as means ± SD. Asterisks (*) on the bar represent statistical significance, *p < 0.05, **p < 0.01, ***p < 0.001 (*N* = 3).

T-cell immune responses, in terms of the percentage of CD4-1^+^, CD4-2^+^, or CD8β^+^ lymphocytes, were also investigated in immunized flounder at days 3, 5, 7, 9, 11, 14, and 28 post-immunization. Lymphocytes in peripheral blood, spleen, and head kidney, were isolated from the immunized fish, and incubated with FCD4-1-Pab, FCD4-2-Pab, FCD8β-Pab antibodies to identify the specific T-cell populations. The fluorescence dot plots obtained from FCM analysis indicate the percentage of CD4-1^+^, CD4-2^+^, or CD8β^+^ lymphocytes determined in one exemplary fish of the three investigated at day 14 post-immunization (Figure 3).

The percentage of CD4-1^+^ T lymphocytes increased significantly at day 7 (*p* < 0.05), peaked on day 11, and then declined to the non-treated level at day 28 in peripheral blood. In both spleen and head kidney the percentage of CD4-1^+^ T lymphocytes increased significantly at days 7 and 9 (*p* < 0.05), respectively, and both reached the maximum level at day 14 (Figure 3A).

In both peripheral blood and head kidney, the proportion of CD4-2^+^ T lymphocytes showed a tendency to initially increase followed by a decrease to normal levels. A significant increase was observed at day 9 (*p* < 0.05), peak levels were reached at day 14, followed by a steady decrease in CD4-2^+^ T lymphocyte levels. In comparison, in spleen these cell levels were dramatically augmented at day 7 (*p* < 0.05), reached their peak at day 11, before decreasing back to normal levels (Figure 3B).

The percentage of CD8β^+^ T lymphocytes showed a marginal increase in peripheral blood and spleen, in spite of the fact that a prominent increase was observed in spleen at day 11 (*p* < 0.05). In head kidney the percentage of CD8β^+^ T lymphocytes were initially increased at day 9 (*p* < 0.05), peaked at day 11, followed by a consistent decrease (Figure 3C).

No significant increase in CD4-1^+^, CD4-2^+^, and CD8β^+^ lymphocytes were detected in the fish transfected with the control plasmid. Thus, it was concluded that the observed increase in T-cell immune responses were induced by the pVAA vaccine.

### pVAA Vaccine Elicits Humoral Immune Response

**Figure 4:**
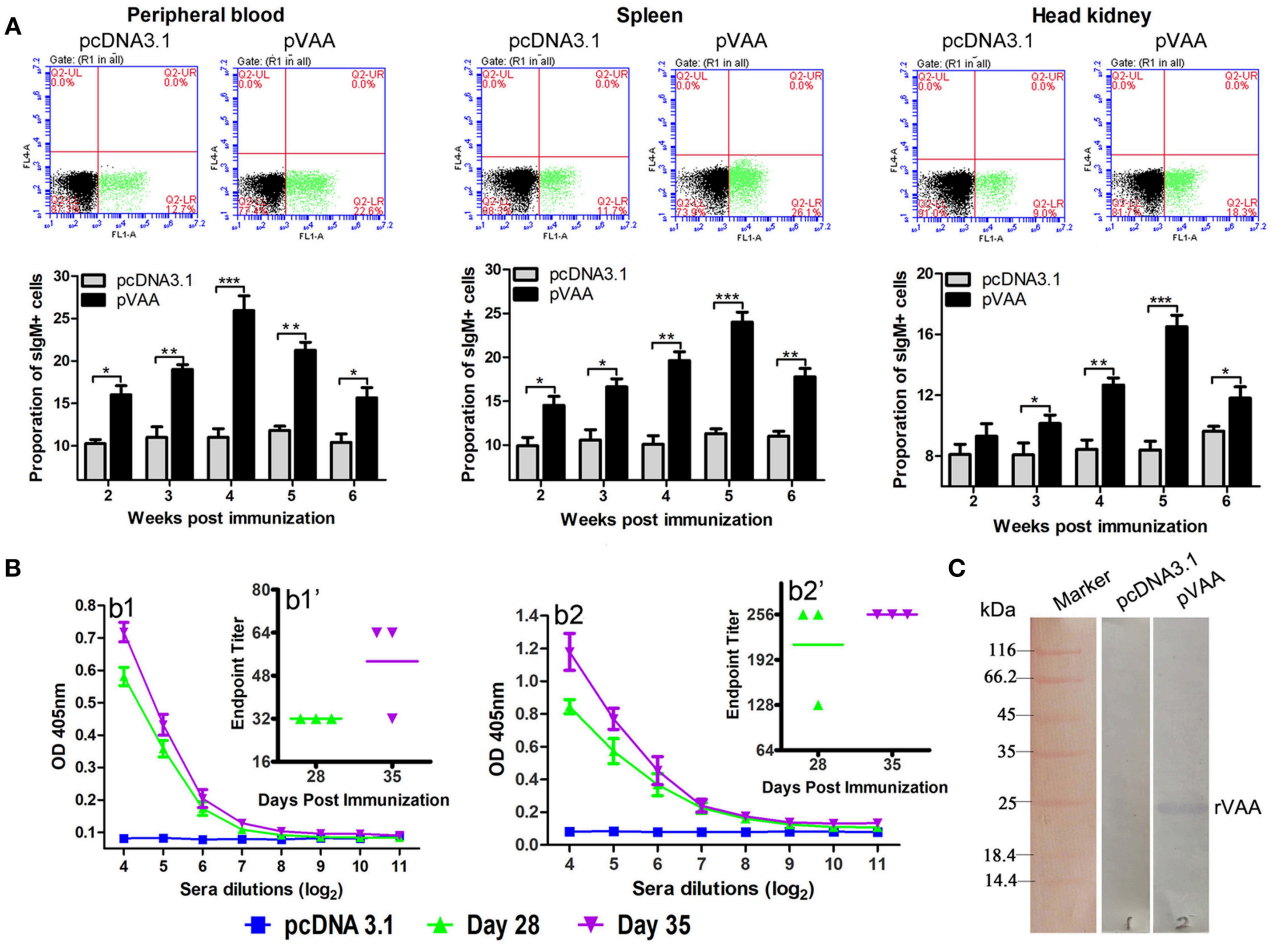
Humoral immune response induced by pVAA vaccination. **(A)** FCM assay investigating the percentage of IgM+ B lymphocytes in peripheral blood, spleen and head kidney of flounder. Plots show the percentage of sIgM^+^ lymphocytes in pcDNA3.1 and pVAA immunized fish at week 5 post-immunization. Lymphocytes were isolated from immune related tissues at week 2, 3, 4, 5, and 6 after injection, and incubated with mouse anti-IgM monoclonal antibody and goat-anti-mouse Ig-FITC antibody to measure the percentages of sIgM^+^ B lymphocytes. All values are shown as means ± SD. Asterisks (*) on the bar represent statistical significance, *p < 0.05, **p < 0.01, ***p < 0.001 (*N* = 3). **(B)** ELISA analysis measuring the specific anti-*V. anguillarum* (b1) or anti-rVAA (b2) antibody production in immunized fish. Binding to *V. anguillarum* or rVAA was analyzed using sera from flounder at different time points (days 28 and 35) post-injection at various dilutions. The data shown are representative of at least three separate experiments. Endpoint binding titers analysis of specific anti-*V. anguillarum* (b1') or anti-rVAA (b2') antibody. **(C)** Western blot analysis of the presence of specific antibodies against rVAA in sera induced by pVAA inoculation. rVAA protein was electrophoresed on a 12% SDS polyacrylamide gel and analyzed by western blot with sera from pVAA immunized fish at day 35 post-vaccination. Sera obtained from pcDNA3.1 immunized controls at day 35 post-vaccination was used as a negative control.

The ability of the pVAA to induce functional humoral immune responses was evaluated by means of specific antibody production and sIgM^+^ B lymphocyte proliferation. Lymphocytes in peripheral blood, spleen, and head kidney were isolated from the immunized fish, and the percentage of sIgM^+^ B lymphocytes was evaluated by FCM as showed in Figure 4A. The fluorescence dot plots indicate the percentage of sIgM^+^ B lymphocytes from one of the three investigated fish at week 5 post-immunization.

Two weeks after inoculation, the percentages of sIgM^+^ lymphocytes showed a significant increase (*p* < 0.05) and reached the peak level at week 4–5 post-immunization, in both peripheral blood and spleen tissues. In head kidney, the percentage of sIgM^+^ lymphocytes increased significantly at week 3 (*p* < 0.05), peaked at week 5, and decreased at week 6 post-immunization. In the control group, the percentage of lymphocytes maintained a consistent level during the experimental period.

Sera were collected from three immunized fish in each group, and then tested by ELISA for specific anti-*V. anguillarum* or anti-rVAA antibodies titers using *V. anguillarum* or rVAA as capture antigens, respectively. A significant induction of anti-*V. anguillarum* or rVAA antibodies was observed on day 28 with a further slight increase noted on day 35 in pVAA vaccinated fish (Figures 4B1,2). To verify that the immune sera could specifically bind to the rVAA protein, we performed western blot analysis and compared the ability of sera from pVAA or pcDNA3.1 immunized fish to detect the rVAA protein. The results revealed that sera from pVAA immunized fish specifically bound to the rVAA protein, while no visible band was observed in the membrane incubated with sera from the control group (Figure 4C). The results of western blot analysis confirm the ELISA results, suggesting that rVAA-specific antibodies were induced in flounder by immunization with the pVAA vaccine.

These data demonstrate that compared to fish inoculated with pcDNA3.1, the use of the pVAA vaccine promoted the production of specific antibodies and resulted in changes in the percentage of sIgM^+^ lymphocytes in response to *V. anguillarum* infection in flounder fish, indicating the elicitation of a humoral immune response.

### pVAA Vaccine Protects Flounder From *V. anguillarum* Infection

**Figure 5:**
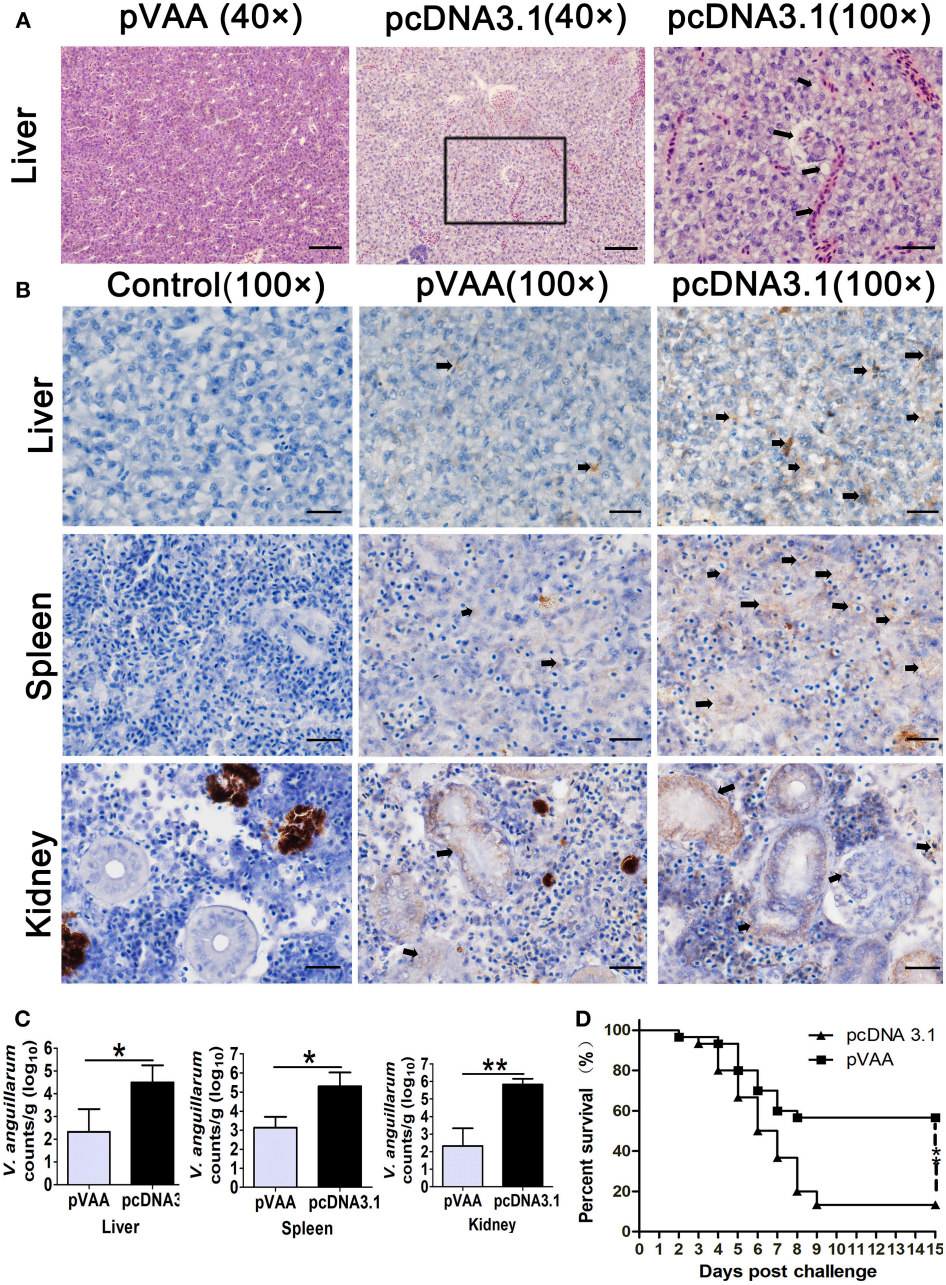
The immune protective efficacy provided by pVAA vaccination. At 7 weeks post-immunization fish were intraperitoneally injected with 1 × 10^7^ CFU *V. anguillarum*. The liver, spleen and head kidney were removed and divided into two sections at day 7 post-challenge: one part of the tissue was fixed and sectioned, for staining with H&E and IHC to examine pathological lesions and bacterial appearance; the other part was stored in RNA later, total RNA extracted and bacterial load examined by qPCR. **(A)** Intramuscular administration of pVAA vaccine inhibited the histopathological changes in liver of fish after *V. anguillarum* infection. Magnification, × 40 and × 100. **(B)** IHC detection of *V. anguillarum* in liver, spleen and head kidney of flounder at day 7 post-challenge. The brown sediment (arrowhead) reveals the presence of *V. anguillarum*. Magnification, ×100. **(C)** Bacterial load in liver, spleen and head kidney of fish. **(D)** Survival percentage of the immunized fish after challenge with *V. anguillarum*.

To determine any gross histopathological changes resulting from *V. anguillarum* infection, fish were sacrificed at day 7 post-challenge, and livers, spleens, and head kidneys collected for H&E staining. H&E stained liver sections exhibited severe lesions in pcDNA3.1 immunized fish following the bacterial challenge. Specifically, numerous erythrocytes penetrated the interstices and large lipid vacuoles appeared in the liver of pcDNA3.1 immunized fish. However, in pVAA vaccinated fish only slight lesions were visualized in the liver (Figure 5A). Moreover, there were no much lesions observed in spleen and head kidney of pVAA or pcDNA3.1 immunized fish (data not shown).

IHC was conducted to verify the presence of *V. anguillarum* in the tissues of the liver, spleen, and head kidney. As shown in Figure 5B, an increase in the staining of *V. anguillarum*-positive cells (arrowhead) was observed in the tissue sample from the pcDNA3.1 vaccinated group compared to pVAA immunized fish, indicating that pVAA may potentially inhibit the colonization of *V. anguillarum*.

To confirm whether the pVAA vaccinated fish were able to suppress the *V. anguillarum* infection, the bacterial burden was measured at day 7 post-challenge by investigating *rpoS* gene concentration in liver, spleen, and head kidney, according to the equation:

$$\begin{array}{l} \text{Y}=-3.1043X+32.15 \end{array}$$

(Supplementary Figure 3, Y = Ct values, X = logarithm of *V. anguillarum* cells).

In general, the bacterial burden appeared to be significantly lower (*p* < 0.05) in the liver and spleen tissues from the pVAA vaccinated flounder compared with the pcDNA3.1 injected group, with a highly significant reduction in the bacterial burden (*p* < 0.01) detected in the kidney of vaccinated fish compared with the control vaccinated fish (Figure 5C). As expected, the *rpoS* gene wasn't detected in mock challenged fish (data not shown).

The potential ability of the pVAA vaccine to affect the RPS of flounder fish following *V. anguillarum* infection was evaluated. After challenge with *V. anguillarum*, dead fish in the pcDNA3.1 immunized group were initially observed at day 2 and subsequently died quickly over days 4–7. The cumulative mortality in the control vaccinated group reached 86.67% at day 15. Comparatively, in the pVAA vaccinated group, deaths were only observed in the initial 7 days post-immunization, with 17 fish surviving until the end of the experimental period. The cumulative mortality rate in pVAA group was 43.33%, thus equivalent to a relative percent survival (RPS) of 50.00% (Figure 5D).

## Discussion

Various forms of vaccine against *V. anguillarum* have been investigated for use in aquaculture, including attenuated vaccines (22), subunit vaccines (23, 24), and DNA vaccines (17, 25). Under laboratory conditions, they have been demonstrated to confer ideal protection for fish. Indeed, an inactive vaccine against *V. anguillarum* has now been commercialized for this use (26). DNA vaccines, in comparison to traditional antigen vaccines, possess advantages in terms of simple and efficient production processes, they are relatively stable and inexpensive to manufacture, and offer the potential induction of both humoral and cellular immune responses. Consequently, they are considered the next generation approach in vaccines (27). In the present study, a recombinant DNA plasmid encoding the *VAA* gene of *V. anguillarum* was constructed as a potential DNA vaccine for the prevention of flounder fish infection.

VAA belongs to a family of transcriptional regulatory factors that play a role in bacterial pathogenicity through the direct regulation of virulent gene expression. In our previous study, VAA was identified by western blotting with fish antibodies. It was demonstrated that the recombinant protein emulsified with Freund's complete adjuvant was proven to induce a humoral immune response in injected fish, conferring a RPS of 78.38% for flounder (6). Considering the previously observed immunoprotective effect provided by rVAA, the recombinant DNA plasmid assessed here may seem less attractive as it resulted in a lower RPS of 50.00%. However, many elements, such as vaccine dose, application of adjuvant, route of administration, and temperature of water, can influence vaccine efficacy (28). It has been shown in fish that a high vaccine dose combined with maintenance of water temperature within the proper range can induce increased immune protection (29, 30). Moreover, Freund's complete adjuvant may trigger early innate defense mechanisms and assist in the generation of robust and long-lasting immune responses to promote protective immunity against pathogens (31). The correct dosing of a DNA plasmid combined with the introduction of genes encoding molecular adjuvants could offer important strategies that may help to optimize the immune protection conferred by pVAA (32).

Promoters that drive gene expression can aid in establishing dosing from a DNA plasmid vaccine. A considerable number of promoters have been investigated for their ability to drive the expression of exogenous genes in aquaculture fish (33, 34). Among these promoters, the immediate early promoter of the cytomegalovirus (CMV), which is the most widely used in DNA vaccines, gives the greatest expression of foreign genes (35). Previous studies have demonstrated that transfected fish cell lines are able to express DNA vaccines when instigated by a mammalian promoter (13, 24, 36, 37). In the present study, the pcDNA3.1 plasmid containing a CMV promoter was used as a vector to construct the recombinant plasmid. RT-PCR analysis of pVAA transfected cells revealed a specific band at 513 bp, corresponding to the *VAA* gene size. IFA further showed that the transfected HINAE cell line acquired the ability to produce rVAA protein. Taken together these results indicate the successful transcription and translation of the *VAA* gene from the transfected plasmid *in vitro*.

Following intramuscular immunization with a DNA vaccine, it has been hypothesized that myocytes could efficiently take up the DNA plasmid and subsequently express the encoded proteins through their transverse tubule system and multinucleate nature (38). Once the protein has been synthesized, it is released from the myocytes either as a secreted protein or due to cell damage/apoptosis, upon which it is recognized and presented by antigen presenting cells (such as macrophages, dendritic cells, and B cells), resulting in the downstream activation of helper and cytotoxic T-cells (39, 40). Expression of transfected viral proteins has been observed by many researchers in myocytes along with the infiltration of lymphocytes and macrophages around the injection site (41–43). In the present study, specific immunofluorescence around the injection site was observed in pVAA immunized fish. It is thus considered highly probable that the VAA-producing cells were myocytes. Indeed, future research will involve confirmation of the cell type responsible for rVAA protein production and investigation of local immune responses at the injection site.

It is established that following intramuscular administration of a DNA vaccine, the expressed antigens are absorbed, processed, and presented by antigen-presenting cells, and recognized by a T-cell receptor via MHCI and MHCII molecules. Binding of MHCI or MHCII receptors on T-cells results in the activation of either a CD8^+^ T-cell (cytotoxic T-cell) or a CD4^+^ T-cell (helper T-cell) immune response, respectively (44). In mammals, numerous cellular studies have confirmed the ability of DNA vaccines to induce a cellular immune response in the control of pathogenic infections (45–48). Indeed, one study showed that the percentage of CD3^+^CD4^+^CD8^−^ and CD3^+^CD8^+^CD4^−^ T-cells identified in the spleen of mice increased in response to DNA vaccines encoding the *GRA17* and *GRA23* genes from *Toxoplasma gondii* (49). To date, cellular immune responses induced by DNA vaccines in fish models have largely been investigated via transcriptional responses. This has been due to the lack of availability of antibodies for markers of specific T-cell subpopulations (50–53). Recently, with the production of specific antibodies for fish T lymphocytes, it has become possible to investigate cellular immune responses at the translational level. This has been exemplified by the investigation of cellular immune responses induced by various antigens in carp and rainbow trout (54–59). In this study, we used FCM analysis of single stained CD4-1^+^, CD4-2^+^, and CD8β^+^ cells to evaluate the cellular immune responses induced by the pVAA vaccine. High percentages of CD4-1^+^, CD4-2^+^, and CD8β^+^ T lymphocytes were observed in the peripheral blood, spleen, and head kidney of fish vaccinated with pVAA compared to the tissues of fish injected with the negative control pcDNA3.1. This is in line with the findings of a previous study indicating that DNA vaccines are able to induce an adaptive cellular immune response, via the upregulation of CD4 and CD8 (60). The proliferation of antigen-specific T-cells in pVAA vaccinated flounder fish following bacterial infection indicates the induction of a specific cellular immune response and demonstrates the potential of this antigen as a vaccine candidate. Correspondingly, a previous report of a viral infection-specific T-cell immune response induced by a DNA vaccine to the spring viremia of carp virus was identified through the proliferation of Zap70^+^ T-cells (42). This opens up the potential of future studies evaluating a possible antigen-specific T-cell response induced by the pVAA DNA vaccine.

Research has shown that antigen-specific antibodies play a role in neutralizing the effects of bacterial toxins and in opsonizing pathogen for elimination by phagocytosis, to prevent pathogenic infections in fish (61). Antigen-specific antibodies are an important parameter of the humoral immune response following vaccination and offer the most reliable correlates with protection, in conferring protective immunity for immunized fish (28). Previous studies have suggested that intramuscular or oral administration of DNA vaccines can lead to the production of antigen-specific antibodies against bacteria and recombinant proteins, with the highest level of antiserum being observed between weeks 5 and 7 post-vaccination (62–67). In the present study, production of specific antibodies against *V. anguillarum* or rVAA was detected at days 28 and 35 post-immunization. Antibody production has been previously shown to be influenced by factors such as fish size and age, water oxygenation and temperature (30, 68), and antigenic dose (60). Within this study the size and age of the selected fish were approximately equal; the oxygenation, water temperature, and other rearing conditions were normalized across all tanks; and the dose of DNA plasmid was also well-controlled. Consequently, any variation in antibody levels between fish would be expected to be low. Nonetheless, a greater sample population of fish used to evaluate serum antibody titers offers increased accuracy in the determination of such titers (69). Accordingly, it is important that future studies ensure sufficient fish numbers are used in order to accurately gauge antibody titer.

Within this study, we demonstrated that immunization with the pVAA vaccine resulted in the induction of a humoral immune response in flounder fish. This was shown by a significant increase in the percentage of sIgM^+^ lymphocytes in the peripheral blood, spleen and head kidney of pVAA vaccinated flounder compared with pcDNA3.1 control immunized fish. A possible explanation for this observation may be that the expression of the VAA protein increases the percentage of sIgM^+^ lymphocytes. Indeed, similar results have been noted in previous studies demonstrating the induction of sIgM^+^ B lymphocytes following intramuscular or oral immunization with DNA plasmids (13, 42, 70). Taken together, these results suggest that the intramuscularly administrated pVAA vaccine induced a humoral immune response in flounder.

The bacterial load present in tissue offers a direct parameter that reflects the number of pathogens present in immunized fish post-challenge, and can be used to evaluate the effective elimination or suppression of a pathogen in organisms protected by a vaccine (20, 21). Quantitative PCR is associated with the advantages of being sensitive, specific, and relatively less time-consuming than other methods, and thus offers an ideal method to assess the bacterial burden colonized in tissues. The *rpoS* gene, which is a housekeeping gene for the general regulation of stress and involved in the pathogenic colonization of host tissues, was selected as the target gene for the detection of *V. anguillarum* in fish (71). In this study, the bacterial burden in spleen, liver, and head kidney of immunized flounder post-challenge was analyzed by a qPCR assay targeting the *rpoS* gene and via IHC. The results revealed that the number of *V. anguillarum* in the tissues of pVAA immunized fish was significantly lower than that of the pcDNA3.1 immunized control group, indicating that pVAA may have induced an immune response to suppress bacterial replication. Following challenge with *V. anguillarum*, the bacteria penetrate and colonize the fish, affecting various organs with a systemic infection (72). In this study, following the infection of flounder, histopathological analysis showed that prior immunization with pVAA reduced the pathological changes observed in liver. Importantly, pVAA conferred protection rates to flounder of ~50%, which can be considered moderate given the fact that the accumulated mortality reached 86.67% in the control pcDNA3.1 immunized group.

Overall, this study not only reveals that pVAA could act as a potential DNA vaccine candidate, but also contributes to the understanding of the humoral and cellular immune responses triggered by DNA vaccination in flounder. Additionally, our findings may offer solutions regarding the establishment of a vaccine efficacy evaluation system, instead of depending solely upon calculating survival rates after pathogen infection.

## Ethics Statement

Investigations were conducted strictly with the ethical standards and the Guidelines of Regulations for the Administration of Affairs Concerning Experimental Animals documented by the State Science and Technology Commission of Shandong Province. These studies were also allowed by the Committee of the Ethics on Animal Care and Experiments at Ocean University of China.

## Author Contributions

JX and HX contributed to the conception and design of this study, performed most of experiments and statistical analysis, drafted and revised the manuscript. XT and XS participated in the design of the study, helped analyzed experiments and data. WZ designed the study, edited the manuscript, and provided reagents and experiment space. All the authors read and approved this version of the final manuscript and confirm the integrity of this work.

### Conflict of Interest Statement

The authors declare that the research was conducted in the absence of any commercial or financial relationships that could be construed as a potential conflict of interest.
