# Supplementary material for: A DNA Vaccine Encoding the VAA Gene of Vibrio anguillarum Induces a Protective Immune Response in Flounder
Source: Front Immunol. 2019 Mar 19;10:499. doi: 10.3389/fimmu.2019.00499 (PMC6435001; doi:10.3389/fimmu.2019.00499)
Supplement: Supplementary file 1 [file Data_Sheet_1.docx]

***Supplementary Material***

**A DNA Vaccine Encoding the VAA Gene of *Vibrio anguillarum* Induces a Protective Immune Response in Flounder**

**Jing Xing^1, 2^, Hongsen Xu^1^, Xiaoqian Tang^1, 2^, Xiuzhen Sheng^1^, Wenbin Zhan^1, 2,^ ***

*** Correspondence:** Wenbin Zhan: [wbzhan@ouc.edu.cn](mailto:wbzhan@ouc.edu.cn)


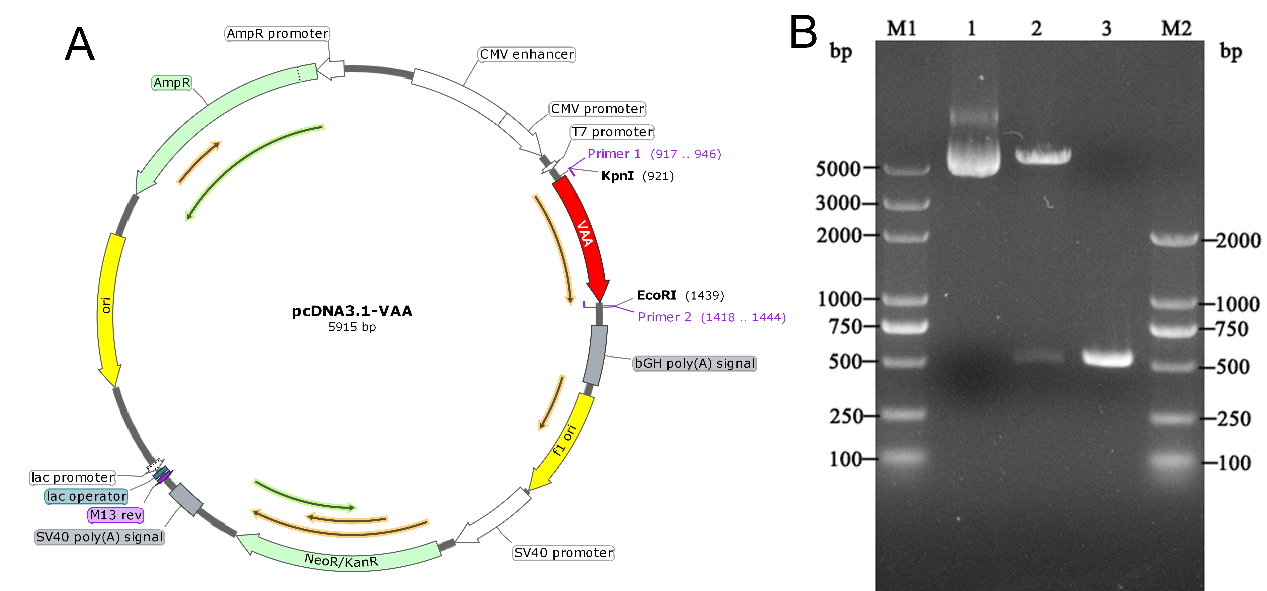


**Supplementary Figure 1. Construction of pcDNA3.1-VAA plasmid.** **(A)** Structure of pVAA vector. The *VAA* gene was inserted into *Kpn* I and *Eoc*R I restriction enzyme site and controlled by CMV and T7 promoter. **(B)** Identification of pVAA plasmid. M1: DL-5000 DNA markers; M2: DL-2000 DNA markers; Lane 2: pVAA plasmid; Lane 2: pVAA plasmid was digested by Kpn I and EocR I; Lane 3: PCR product of VAA gene amplified from pVAA plasmid.


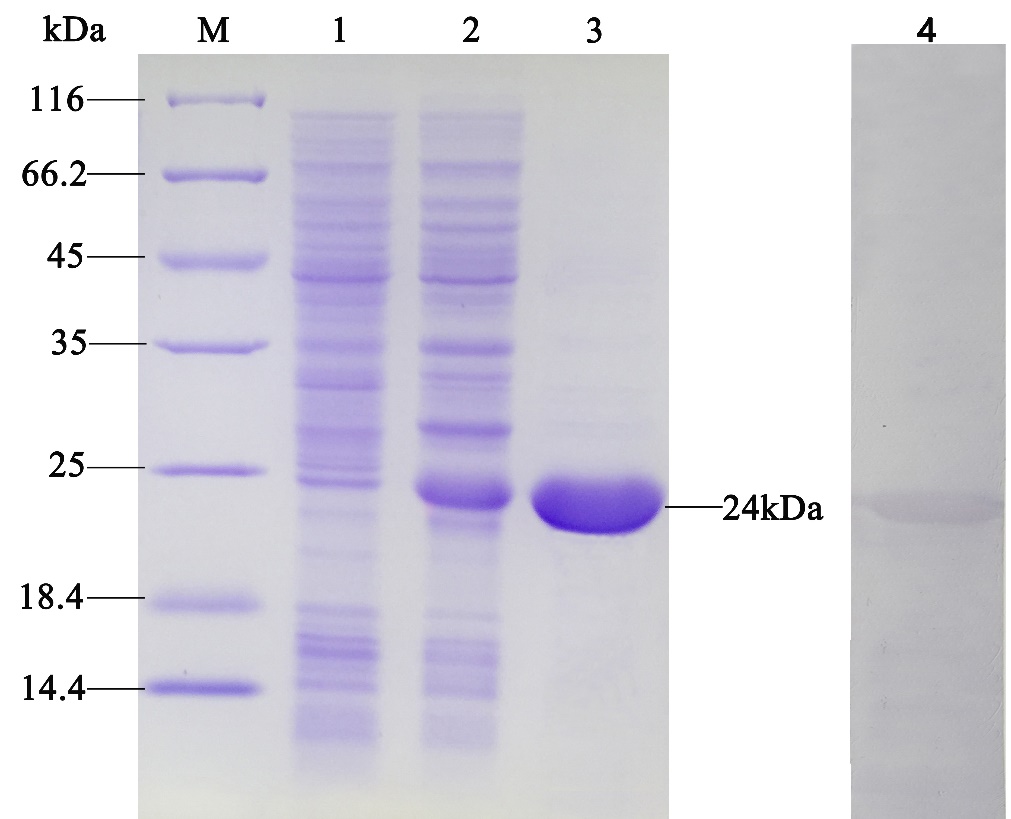


**Supplementary Figure 2. The SDS-PAGE and Western blot results of recombinant VAA proteins.** M: Molecular mass marker; Lane 1: Recombinant *E. coli* BL21 without IPTG induction; Lane 2: Recombinant *E. coli* BL21 induced with IPTG; Lane 3: Purified VAA protein; Lane 4: Purified VAA protein analyzed by western-blot.


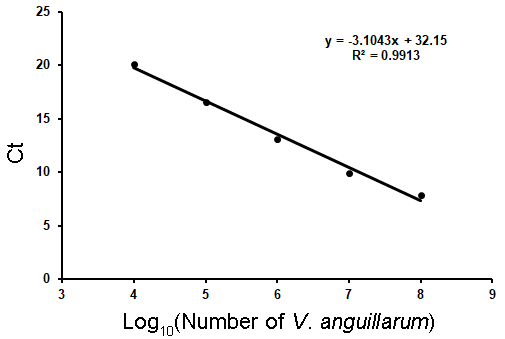


**Supplementary Figure 3. The standard curve generated from the Ct values (y) against log10 (number of *V. anguillarum* cells (x)).**
